# Silent Outbreaks of *Candida duobushaemulonii* in a Pediatric Ward in Brazil

**DOI:** 10.3390/antibiotics15030237

**Published:** 2026-02-25

**Authors:** Daniel Wagner de Castro Lima Santos, Bram Spruijtenburg, Eelco F. J. Meijer, Dayse Azevedo Coelho de Souza, Conceição de Maria Pedrozo e Silva de Azevedo, Jacques F. Meis

**Affiliations:** 1 Instituto D’Or de Pesquisa e Ensino (IDOR), São Luís 65076-820, MA, Brazil; danielinfectologista@gmail.com; 2 Hospital Universitário, Universidade Federal do Maranhão, São Luís 65020-070, MA, Brazil; 3 Department of Medical Microbiology, Radboud University Medical Center, 6525 GA Nijmegen, The Netherlands; b.spruijtenburg@cwz.nl (B.S.); eelco.meijer@cwz.nl (E.F.J.M.); 4 Radboudumc-CWZ Center of Expertise for Mycology, 6532 SZ Nijmegen, The Netherlands; 5 Department of Medical Microbiology and Immunology, Canisius-Wilhelmina Hospital/Dicoon, 6532 SZ Nijmegen, The Netherlands; 6 Programa de Pós Graduação em Ciências da Saúde, Universidade Federal do Maranhão (UFMA), São Luís 65020-070, MA, Brazil; coelho.dayse@gmail.com (D.A.C.d.S.);; 7 Hospital de Cancer Aldenora Bello(HCAB), São Luís 65031-630, MA, Brazil; 8 Institute of Translational Research, Cologne Excellence Cluster on Cellular Stress Responses in Aging-Associated Diseases (CECAD) and Excellence Center for Medical Mycology, University of Cologne, 50923 Cologne, Germany

**Keywords:** fungal outbreak, rare yeast, *Candida duobushaemulonii*, *Candida haemulonii* species complex, whole genome sequencing, antifungal susceptibility

## Abstract

**Background**: While *Candida auris* is well known to cause hospital outbreaks, other species in the *C. haemulonii* complex are less well documented but gained attention as opportunistic pathogens. Only one documented outbreak has been published. We describe the second, silent, fungemia outbreak due to antifungal-susceptible *C. duobushaemulonii*. **Methods**: We retrospectively genotyped six *C. duobushaemulonii* bloodstream isolates, collected in a 4-month-period in 2022 (n = 4) and during a week in 2024 (n = 2) in pediatric patients in Brazil. Whole genome sequencing (WGS) was done and compared to n = 33 publicly available genomes, including four cases from an outbreak in Panama. Antifungal susceptibility was performed with the reference CLSI method. **Results**: MALDI-TOF-MS identified isolates as either *C. pseudohaemulonii* or *C. duobushaemulonii* albeit with low scores. ITS sequence analyses confirmed all isolates as *C. duobushaemulonii*. WGS proved the presence of an outbreak among four pediatric patients in 2022 and a genetically distinct cluster of two cases in 2024. All six isolates were susceptible to azoles and echinocandins and were interpreted as being resistant to amphotericin B with a MIC at breakpoint of 2 µg/mL. **Conclusions**: This study describes the second documented outbreak due to the rare yeast *C. duobushaemulonii*, belonging to the *C. haemulonii* species complex, during 2022–2024 in patients admitted to a pediatric oncology ward in a Brazilian hospital.

## 1. Introduction

The recent emergence of *Candida auris*, a global fungal public health threat [1], has drawn attention to seven closely related, less well-known species in the *C. haemulonii* species complex that include *C. haemulonii sensu strictu*, *C. duobushaemulonii*, *C. pseudohaemulonii*, *C. vulturna*, *C. haemulonii* var. *vulnera* [2,3], and the recently identified species *C. khanbhai* [4] and *C. molenica* [5]. Invasive infections other than *C. auris* are uncommon and are mainly encountered in India, China and the Americas, including sporadic nosocomial outbreaks involving *C. haemulonii*, *C. duobushaemulonii* and *C. vulturna* [3]. *C. duobushaemulonii*, previously classified as *C. haemulonii* group II, was re-categorized as a separate species in 2012 [6]. While *C. haemulonii* was already described in 1962 [7], *C. duobushaemulonii*, the second species in the *C. haemulonii* species complex, was retrospectively found in a yeast culture collection at the US Centers for Disease Control (CDC) as early as 1990 [8]. Although cases of *C. duobushaemulonii* infection have been rare to date, they have been mainly reported in the USA, Latin America, India, and China [8,9,10,11,12,13]. These studies either mentioned no clinical background or included only adults in the study population except one study which had three pediatric patients among 10 cases with fungemia [9]. A study on rare fungemias in children in Taiwan reported only one non-oncologic patient with *C. duobushaemulonii* among 46 cases [14]. *C. duobushaemulonii* has several virulence factors in common with *C. auris* which aid in medical-device colonization, antifungal and disinfectant tolerance, osmotic stress and thermotolerance, all contributing to its persistence in hospital environments and risks of nosocomial outbreaks [15,16].

A series of invasive *C. duobushaemulonii* infections were identified in Panama in 2016 concomitant with cases of *C. auris* [8,9,17] and subsequent epidemiological investigations, including whole genome sequencing (WGS), confirmed the first outbreak with this sibling of *C. auris* in 2020 [8]. By using high-resolution WGS analysis and a large set of publicly available genomes, we aimed to study two in-hospital transmission events with *C. duobushaemulonii* in a pediatric oncology ward in Northeast Brazil, where earlier also an outbreak with *C. vulturna* occurred [18].

## 2. Results

### 2.1. Patients and Isolate Identification

The clinical and laboratory parameters of the six patients are summarized in Table 1 and Table 2. The mean age of the patients was 6.6 years (range 3 to 12 years), and 50% were male. The majority suffered from acute lymphoblastic leukemia as an underlying condition. A central venous catheter was present in all patients at the time of diagnosis, and two patients received total parenteral nutrition. Five patients were treated with broad-spectrum antimicrobial therapy at the time of fungemia. Fever and chills were the most common presenting features and were observed in five patients (83%). Concomitant bacteraemia was detected in two patients, involving *Acinetobacter baumannii* in one case and *Staphylococcus epidermidis* in the other. Severe neutropenia (<100 cells/mm^3^) was present in two patients at the time of diagnosis of fungemia. All patients remained haemodynamically stable during targeted antifungal therapy with intravenous fluconazole (12 mg/kg followed by 6 mg/kg once daily) and follow up blood cultures remained negative. No cases of deep-seated infections were detected. The 30-day mortality was 67% (4/6 patients), most likely related to the severe underlying diseases. We suspected an outbreak because the first (2022) and second (2024) group of patients were timely restricted and had the same provisional pathogen isolated. The first outbreak occurred in the same period as a larger outbreak with *C. vulturna*. We considered this outbreak to be silent because it was only uncovered several months after the event.

**Figure 1:**
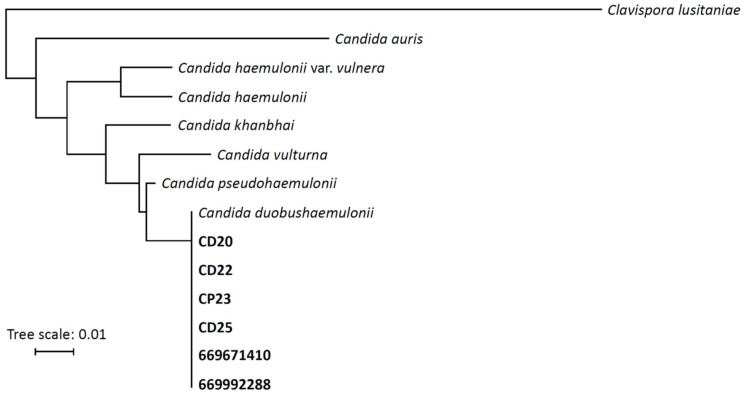
Phylogenetic tree based on the ITS region of *Candida haemulonii* species complex including *Clavispora lusitaniae* as outgroup. Isolates from the current study are marked in bold.

The six clinical isolates were initially misidentified as *C. pseudohaemulonii/C. duobushaemulonii* (with low scores of <1.7) or retrieved no database match with MALDI-TOF-MS but were later determined to be *C. duobushaemulonii* confirmed by sequencing of the ITS region (Figure 1).

### 2.2. Outbreak Investigation

**Figure 2:**
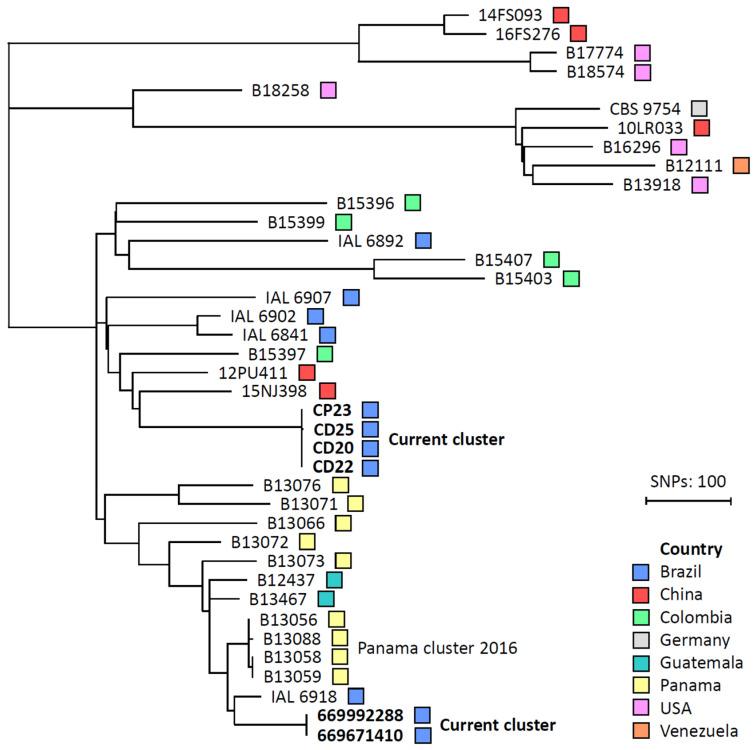
Whole genome sequencing (WGS) single nucleotide polymorphism (SNP) analysis of C. duobushaemulonii isolates. On 33 publicly available WGS data, SNP analysis was performed. Isolates from this study (2022–2024) are marked in bold. B09383 was used as the reference genome, and positions with a minimal coverage of 15 were included for the comparative analysis and the tree was constructed with the neighbor-joining method using MEGA11 (v11.0.10) and iTOL (v6) software.

All isolates were sent for Illumina sequencing and results are detailed in Supplementary Table S1. By conducting WGS SNP analysis on all six *C. duobushaemulonii* blood culture isolates and 33 controls from eight countries, an overall low genetic diversity was found with less than 1500 SNPs difference. The six isolates from the current investigated hospital clustered in two distinct branches, a tight cluster of four isolates occurring in 2022 and one cluster in 2024 respectively (Figure 2). Within the two groups, isolates were virtually identical (2 SNPs difference at most), while between isolates of the two identified outbreaks the variation was 462 SNPs. When compared to the nearest control isolates for both clusters, the genetic distance was at least 100 SNPs. A cluster of four isolates, from different patients, from a published outbreak in Panama formed a cluster on the phylogenetic WGS tree, with two identical isolates (0 SNPs) and two with less than 5 SNPs difference. No aneuploidy or large-scale copy number variation was found in the six isolates involved in the current outbreaks. By including control isolates from other countries, there were no monophyletic branches visible based on the country or continent of origin.

### 2.3. Resistance Investigation

By conducting AFST according to CLSI microbroth dilution, isolates were found to be resistant to amphotericin B (2 µg/mL) and susceptible for fluconazole (4–8 μg/mL) and echinocandins (≤0.125 μg/mL) (Table 3). Genomic inspection of known, resistance-associated genes *ERG3*, *ERG6*, *ERG11* and *FKS1* did not show any missense mutations when compared to the reference genome.

## 3. Discussion

*C. duobushaemulonii*, previously classified as *C. haemulonii* group II, was re-categorized and named a separate species in 2012 [6], as part of the *C. haemulonii* species complex that also comprises *C. haemulonii senso strictu*, *C. haemulonii* var. *vulnera*, and *C. pseudohaemulonii* [2]. Retrospective studies of archived isolates found that the earliest recorded *C. duobushaemulonii* were from 1990, stored in the CDC culture collection [8], and from 1996 in Spain [19]. *C. duobushaemulonii* is an opportunistic yeast that has been identified in various environments and from clinical settings such as deep cutaneous infections [20], recurrent vulvovaginal candidiasis [21], chronic wound infections [22] and candidemia [9]. Additionally, antifungal susceptibility testing has shown that *C. duobushaemulonii* is often resistant to amphotericin B and fluconazole [6,9]. Most cases of *C. duobushaemulonii* infections have been reported in the USA, Latin America and Asia [8,9,10,11,12,13] with only sporadic reports from other countries suggesting a geographic restriction. As seen with many other rare *Candida* species, *C. duobushaemulonii* infections are likely underreported because identifying closely related species in the complex is challenging. Phenotypic identification systems can misidentify *C. duobushaemulonii* as closely related species in the *C. haemulonii* species complex. Although MALDI-TOF-MS databases have improved the ability to differentiate *C. auris* and closely related species, *C. duobushaemulonii* is sometimes misidentified as *C. haemulonii* or *C. pseudohaemulonii* [23], as was the case in this report.

A recent review [24] reported that China and especially Brazil were the countries with the highest numbers of *C. haemulonii* species complex infections including *C. duobushaemulonii*. The prevalence rates of the *C. haemulonii* species complex in 12 Brazilian medical centers rose, in the period 2008 to 2013, from 0.9% to 1.7% (2014 to 2019) [25]. A more recent survey of bloodstream infections caused by rare Sacharomycetales in 28 medical centers across Brazil, showed that *C. haemulonii sensu stricto* (14%) and *C. duobushaemulonii* (12%) made up more than 25% among rare isolates. Stratified by two eight-year periods, *C. duobushaemulonii* increased by 400% between the years 2007–2015 versus 2016–2023 [26].

In the most recent Brazilian study, *C. duobushaemulonii* had an increased fluconazole MIC_90_ of 16 µg/mL, and a high amphotericin B MIC_90_ of ≥8 µg/mL similar as earlier data from Brazil [27,28]. A study from the CDC of n = 55 *C. duobushaemulonii* from Latin America showed that 93% had elevated MICs of amphotericin B and, similar as the Brazilian studies, 87% had fluconazole MICs of ≤16 μg/mL [8]. Interestingly, n = 5 isolates with an MIC of 256 μg/mL had the *Y132F* mutation in the *ERG11* gene (*ERG11^Y132F^*), with concomitant elevated voriconazole MICs of 1–2 μg/mL. All abovementioned studies used CLSI methods and thus were comparable with our results. The isolates responsible for the two outbreaks in our study had slightly elevated MICs of amphotericin B and fluconazole. Chromosomal duplications linked to resistance and resistance-associated genes were inspected, which showed wildtype *ERG6* and *ERG11*, which is in line with earlier reports and suggests that susceptibility patterns may have regional variability. In contrast, the first outbreak described in Panama was caused by a fluconazole resistant clone exhibiting *ERG11^Y132F^*, a similar substitution recently reported in China [16]. In contrast, outbreaks in two other Brazilian states with *C. auris*, the most prominent species in the complex, also showed low azole and amphotericin B MICs for clades I and IV, which are known for their resistant phenotype [29,30]. A similar observation was made for *C. vulturna,* another species in the complex, also showing low MICs of fluconazole and amphotericin B [18]. The difference between Brazil and other countries regarding the pressure for developing resistance for these antifungals remains unclear. Unfortunately, no environmental isolates could be retrieved, making the source of the current and previous outbreak unknown [18]. Source tracking is notoriously difficult especially for outbreaks with rare yeasts, although medical devices and contaminated hands of health care workers are occasionally involved [3].

Whole-genome sequencing SNP analysis demonstrated two clusters of clonally related *C. duobushaemulonii* blood isolates within a pediatric hospital in northeast Brazil which had previously reported an outbreak of *C. vulturna* [18]. By comparing the present outbreaks with published *C. duobushaemulonii* WGS data from eight countries, an overall low genetic diversity was found with less than 1500 SNPs, which is concordant with previous reports [8]. Interestingly, the same is also found for related species in the complex, like *C. haemulonii*, *C. vulturna,* or within the different *C. auris* clades [8,31], suggesting that the common ancestors of these populations are a recent expansion within the human population. Within the two clusters in the present outbreak, isolates were virtually identical (2 SNPS at most) and between the clusters from 2022 and 2024 there was a >450 SNP difference. Despite a two-year difference between the two outbreaks in the same hospital, this genetic difference of more than 450 SNPs is unlikely to be accrued within this time period, suggesting two separate introductions and nosocomial transmission events. To date, only one other, retrospectively identified, clonal transmission event for *C. duobushaemulonii* has been reported, consisting of four fluconazole resistant isolates, exhibiting *ERG11^Y132F^*, in a single Panamanian hospital. The latter cluster consisted of an identical blood and nail isolate (0 SNPs), and two blood isolates separated by <5 SNPs [8].

The hospital in Panama recorded n = 14 cases of *C. duobushaemulonii* between November 2016–May 2017 [9], but an outbreak was only confirmed for four cases [8]. Concomitant infections were observed, overlapping in time with other species in the *C. haemulonii* species complex and *C. auris* [8,17], similar as the observed clusters of *C. duobushaemulonii* and *C. vulturna* in Brazil [18]. It is not unimaginable that patients had undetected mixed fungemia with more than one species from the *C. haemulonii* species complex. This has been described at least once in a patient repatriated from Vietnam to Australia with a double infection/colonization with *C. duobushaemulonii* and *C. auris* [32].

The main limitation of this study was the retrospective nature and the failure to find potential environmental sources of the outbreak. Another limitation of this research is the lack of benchmarking data. With only one previous outbreak described in the literature [8]—and without a specified setting—the ability to compare our results remains limited. Consequently, this study should be viewed as an exploratory step in understanding the transmission dynamics of this poorly described sibling of *C. auris*.

## 4. Materials and Methods

### 4.1. Study Population and Isolates

The outbreak occurred in Hospital de Cancer Aldenora Bello, a reference center in São Luís, Maranhão, which is a 150-bed specialized teaching hospital that focuses on oncology. The hospital admits approximately 500 pediatric cancer patients annually. Six pediatric patients were identified with bloodstream infection caused by *C. duobushaemulonii.* Clinical and laboratory patient information was retrospectively collected from chart reviews. Four cases occurred over a three-month period in 2022, and two additional cases were identified two years later, during a one-month period in 2024. Yeasts were isolated from blood cultures taken from febrile patients and processed with the BACTEC 9240 system (Becton Dickinson, Franklin Lakes, NJ, USA).

### 4.2. Species Identification

Isolates were processed for identification with matrix-assisted laser desorption/ionization time-of-flight mass spectrometry (MALDI-TOF-MS) (MALDI Biotyper version 3.1, database MBT 7854 MSP library RUO; Bruker Daltonik, Bremen, Germany). Spectra were obtained from cultures grown for 48 h on Sabouraud agar. A protein extraction protocol with sequential ethanol, formic acid, and acetonitrile was used [33]. All extracts were overlaid with 2 μL of matrix (saturated solution of α-cyano-4-hydroxycinnamic acid in 50% acetonitrile–2.5% trifluoroacetic acid; Bruker Daltonik) and air dried at room temperature to acquire spectra. A score of ≥2 equaled identification to the species level, and a score between 1.7 and 1.9 means identification to the genus level. Final species identification was obtained by sequencing of the ITS2 region with ITS-1 and ITS-4 primers as previously described [8]. DNA of fresh colonies was extracted using the MagNA Pure 96 instrument and the MagNA Pure DNA and Viral NA Small Volume Kit polymerase (Roche Diagnostics, Mannheim, Germany) according to manufacturer’s protocol. In short, the ITS2 region was amplified with a thermocycler (Biometra, Westburg, Germany) using 1 U FastStart Taq polymerase (all Roche Diagnostics, Mannheim, Germany), 1 × FastStart Taq polymerase buffer, MgCl_2_ (3 mM) polymerase (all Roche Diagnostics), forward and reverse primers (5 µM) and isolated DNA. Amplicons were purified using the Ampliclean protocol (NimaGen, Nijmegen, The Netherlands). Sanger sequencing was conducted on a 3500XL genetic analyzer (Applied Biosystems, Foster City, CA, USA). Control sequences of the Metschnikowiaceae clade were retrieved from the National Center for Biotechnology Information (NCBI) nucleotide database and included *C. vulturna* CVDH05 (OQ519941.1), *C. duobushaemulonii* (JX459666.1), *C. pseudohaemulonii* (NR_163771.1), *C. haemulonii* (AY500375.1), *C. haemulonii* var. *vulnera* (JX459686.1), *C. auris* (PP178707.1), *C. khanbhai* (OP626788.1), and *C. lusitaniae* (AF172262.1). Sequences were aligned and a phylogenetic tree was built and visualized as described earlier [8]. Generated ITS sequences of the current study were deposited to the NCBI Genbank database (Accession numbers: PX844698-PX844703).

### 4.3. Antifungal Susceptibility Testing (AFST)

In vitro AFST according to Clinical and Laboratory Standards Institute (CLSI) M27 standard was performed for amphotericin B (Bristol Meyers Squibb, Rotterdam, The Netherlands), fluconazole (Sigma-Aldrich, Amsterdam, The Netherlands), itraconazole (Janssen Cilag, Breda, the Netherlands), voriconazole (Pfizer, New York, NY, USA), posaconazole (Merck, Darmstadt, Germany), isavuconazole (Basilea Pharmaceutica, Basel, Switzerland), anidulafungin (Astellas Pharma, Tokyo, Japan) and micafungin (Astellas Pharma) [34]. Briefly, colonies were diluted in RPMI medium to a final concentration of 1 × 10^3^–5 × 10^5^ CFU/mL obtained with a Genesys 20 Spectrophotometer (Thermo Fisher, Waltham, MA, USA). Microbroth dilution plates were read visually after incubation at 35 °C for 24 h. Minimum inhibitory concentrations (MICs) were defined as the lowest antifungal concentration that reduced growth by 50% or more when compared to the growth control, except for amphotericin B with 100% growth reduction. *Candida parapsilosis* ATCC 22019 and *Candida krusei* ATCC 6258 were used as quality control strains. For many rare yeasts, including *C. duobushaemulonii*, no breakpoints are available. Therefore MICs were interpreted with tentative CDC breakpoints that were established for Candida auris (https://www.cdc.gov/candida-auris/hcp/laboratories/antifungal-susceptibility-testing.html, accessed on 18 February 2026) with the resistance breakpoint for amphotericin B of ≥2 µg/mL, for fluconazole of ≥32 µg/mL and echinocandins (anidulafungin or micafungin) of ≥4 µg/mL.

### 4.4. Whole Genome Sequencing (WGS) Single Nucleotide Polymorphism (SNP) Analysis

All isolates were cultured at 35 °C for 24 h on Sabouraud dextrose agar (SDA) (Oxoid, Hampshire, UK). DNA was extracted and purified with the MagNA Pure 96 systems according to manufacturers’ instructions as described earlier [35]. Next, extracted DNA was treated with 5 µg/µL of RNase A (Merck KGaA, Darmstadt, Germany) at room temperature for one hour, following a second purification with the MagNA Pure 96 system. Final DNA concentration was assessed with Qubit 3.0 Fluorometer (Thermo Fisher Scientific, Waltham, MA, USA) using the double DNA (dsDNA) high-sensitivity option. Genomic libraries were prepared and sequenced with the Illumina Novaseq 6000 platform (Illumina, San Diego, CA, USA) with two times 150 bp paired-end-read mode at Eurofins Genomics (Ebersberg, Germany). Genomic reads were aligned to the *C. duobushaemulonii* reference genome B09383 (GCA_002926085.1) and a SNP analysis was conducted as previously described [36]. Briefly, read alignment was performed using BWA v0.7.19 and generated BAM files were filtered based on the MAPQ score and if reads were properly paired. Variants were called using Freebayes v1.3.9 and subsequently filtered in a combined VCF file using a minimum QUAL score of 100 and a minimum read depth of 20 for each isolate. The phylogeny was visualized with MEGA11 v11.0.10 by constructing a neighbor-joining tree and iTOL v6. Previously, this SNP calling pipeline was validated for C. auris by using a benchmark dataset of clade I isolates [35]. Up to five randomly selected control isolates per country from the National Center for Biotechnology Information (NCBI) Sequence Read Archive (SRA) were included in the SNP analysis, in addition to four Panamanian isolates involved in an earlier outbreak [8] (Supplementary Table S2). Aneuploidy and large-scale copy number variation was assessed with QualiMap BamQC v2.3. With BLAST (https://blast.ncbi.nlm.nih.gov/Blast.cgi, accessed on 23 May 2025) antifungal resistance-associated genes *ERG11* (CXQ87_003531), *FKS1* (CXQ87_002203), *ERG6* (CXQ87_004088), *ERG3* (CXQ87_003761) were located in the B09383 reference genome and isolates were visually inspected for missense mutations using Integrated Genomics Viewer. Missense mutations were defined as position for which isolates had a minimum read depth of 15 times, with a nucleotide variant present in more than 90% of the reads. Raw read data generated during this study have been submitted to the NCBI SRA database under BioProject ID PRJNA1279651.

## Figures and Tables

**Table 1:** Clinical characteristics and factors associated with invasive *Candida duobushaemulonii* infection in a pediatric ward.

Patient	Date of Fungemia	Age/Gender	Cancer Diagnosis	Central Vascular Catheter-Days	Indwelling Urethral Catheter-Days	Nasoenteric Tube	Parenteral Nutrition-Days	Fever (>37.8C)	Chills	Hypotension (Mean Arterial Pressure ≤ 90 mmHg)	Tachycardia (>100 bpm)
CD20	04/25/22	7/M	ALL-B *	PICC *** 30 days	No	No	No	Yes	Yes	Yes	No
CD22	03/27/22	4/F	ALL-B *	PICC *** 32 days	No	No	No	Yes	Yes	Yes	Yes
CD23	04/04/22	10/F	ALL-B *	PICC *** 62 days	No	No	No	No	Yes	No	No
CD25	02/14/22	12/M	ALL-B *	PICC *** 69 days	No	No	Yes/13 d	Yes	No	No	Yes
669992288	05/07/24	4/M	Retroperitoneal embryonic rhabdomyosarcoma (lung as primary focus)	DL-CVC **** 14 days	Yes/90 d	Yes/30 d	Yes/20 d	Yes	Yes	No	Yes
669671410	06/08/24	3/F	ALL-T **	PICC *** 21 days	No	No	No	Yes	Yes	Yes	Yes

* Acute lymphoblastic leukemia of B cells, ** Acute lymphoblastic leukemia of T cells, *** Peripherally inserted central catheter, **** Double-lumen central venous catheter.

**Table 2:** Laboratory data, treatment and outcome of invasive *Candida duobushaemulonii* infection in a pediatric ward.

Patient	Neutrophils/mm^3^ *	Kidney Dysfunction *	Concomitant Bacteremia *	Echocardiography *	Antifungal Therapy	Persistent Candidemia	30 Day-Outcome
CD20	1476	No	No	No	FLU **	No	death
CD22	31	No	*S.epidermidis*	Yes-No vegetation	FLU	No	alive
CD23	1071	No	No	No	FLU	No	alive
CD25	30	No	No	Yes-No vegetation	FLU	No	death
669992288	20,939	No	*Acinetobacter baumannii*	Yes-No vegetation	FCZ-switched to MFG ***	No	death
669671410	411	No	No	No	FLU-switched to AMB ****	No	death

* at the time of fungemia, ** Fluconazole, *** Micafungin, **** Amphotericin B.

**Table 3:** Minimum inhibitory concentrations (MICs) of six *Candida duobushaemulonii* isolates from two outbreaks against eight antifungals according to Clinical and Laboratory Standards Institute (CLSI) M27 guideline.

Lab ID	AMB	FLU	ITC	VOR	POS	ISA	AFG	MFG
CD20	2	4	0.063	0.063	0.031	0.031	0.063	0.063
CD22	2	4	0.063	0.063	≤0.016	≤0.016	0.125	0.063
CP23	2	4	0.063	0.063	≤0.016	≤0.016	0.063	0.063
CD25	2	4	0.063	0.063	0.031	0.031	0.063	0.063
669992288	2	8	0.063	0.063	0.031	0.031	0.063	0.125
669671410	2	4	0.125	0.125	≤0.016	0.031	0.125	0.063

AMB, amphotericin B; FLU, fluconazole; ITC, itraconazole; VOR, voriconazole; POS, posaconazole; ISA, isavuconazole; AFG, anidulafungin; MFG, micafungin. All MICs are in µg/mL.

## Data Availability

The raw WGS read data have been submitted to the NCBI Sequence Read Archive (BioProject ID: PRJNA1279651) and ITS sequences have been submitted to the NCBI Genbank database (Accession numbers: PX844698-PX844703).

## Supplementary Materials

The following supporting information can be downloaded at: https://www.mdpi.com/article/10.3390/antibiotics15030237/s1, Table S1: Overview of sequencing results for the six *C. duobushaemulonii* isolates collected in the current study. Table S2: *C. duobushaemulonii* control isolates (n = 33) used in single nucleotide polymorphism (SNP) analysis.

## Abbreviations

The following abbreviations are used in this manuscript:

AFST	Antifungal susceptibility testing
CDC	US Centers for Disease Control
CLSI	Clinical and Laboratory Standards Institute
ITS	Internally Transcribed Spacer
IV	Intravenous
MALDI-TOF-MS	Matrix-assisted laser desorption/ionization-time of flight mass spectrometry
MIC	Minimum inhibitory concentration
NCBI	National Center for Biotechnology Information
SNP	Single-nucleotide polymorphism
WGS	Whole genome sequencing
