# Supplementary material for: Silent Outbreaks of Candida duobushaemulonii in a Pediatric Ward in Brazil
Source: Antibiotics (Basel). 2026 Feb 25;15(3):237. doi: 10.3390/antibiotics15030237 (PMC13023860; doi:10.3390/antibiotics15030237)
Supplement: Supplementary file 1 [file antibiotics-15-00237-s001.zip › antibiotics-4153765-supplementary.pdf]

Supplementary Tables S1 and S2

Table S1. Overview of sequencing results for the six *Candida duobushaemulonii* isolates collected in the current study that were aligned against the B09383 (GCA_002926085.1) reference genome.

Isolate ID	Average coverage	% of genome covered	Average MAPQ score
CD20	56.9	99.4	58.3
CD22	49.2	99.1	59.0
CP23	53.7	98.8	58.7
CD25	51.3	99.3	58.5
669992288	55.1	99.2	59.2
669671410	57.0	99.5	59.1

Table S2. *Candida duobushaemulonii* control isolates (n=33) used in single nucleotide polymorphism (SNP) analysis

ID	Biosample	SRA	Country
10LR033	SAMN30966967	SRR24294488	China
12PU411	SAMN30966966	SRR24294487	China
14FS093	SAMN30966968	SRR24294489	China
15NJ398	SAMN30966970	SRR24294491	China
16FS276	SAMN30966969	SRR24294490	China
B12111	SAMN14087096	SRR11091981	Venezuela
B12437	SAMN14087088	SRR11091990	Guatemala
B13056	SAMN14087075	SRR11091940	Panama
B13058	SAMN14087077	SRR11091938	Panama
B13059	SAMN14087078	SRR11091937	Panama
B13066	SAMN14087080	SRR11091999	Panama
B13071	SAMN14087081	SRR11091998	Panama
B13072	SAMN14087082	SRR11091996	Panama
B13073	SAMN14087083	SRR11091995	Panama
B13076	SAMN14087084	SRR11091994	Panama
B13088	SAMN14087085	SRR11091993	Panama
B13467	SAMN14087089	SRR11091989	Guatemala
B13918	SAMN33435051	SRR23610455	USA
B15396	SAMN14087090	SRR11091988	Colombia
B15397	SAMN14087091	SRR11091987	Colombia
B15399	SAMN14087092	SRR11091985	Colombia

B15403	SAMN14087093	SRR11091984	Colombia
B15407	SAMN14087094	SRR11091983	Colombia
B16296	SAMN33435052	SRR23610453	USA
B17774	SAMN33435053	SRR23610452	USA
B18258	SAMN33435054	SRR23610451	USA
B18574	SAMN33435055	SRR23610454	USA
CBS 9754	SAMN33783115	SRR23882033	Germany
IAL 6841	SAMN33435047	SRR23610459	Brazil
IAL 6892	SAMN33435045	SRR23610461	Brazil
IAL 6902	SAMN33435046	SRR23610460	Brazil
IAL 6907	SAMN33435044	SRR23610462	Brazil
IAL 6918	SAMN33435048	SRR23610458	Brazil
